# Autophagic control of cell ‘stemness’

**DOI:** 10.1002/emmm.201201999

**Published:** 2013-03-05

**Authors:** Huize Pan, Ning Cai, Mo Li, Guang-Hui Liu, Juan Carlos Izpisua Belmonte

**Affiliations:** 1 National Laboratory of Biomacromolecules, Institute of Biophysics, Chinese Academy of SciencesBeijing, China; 2 Gene Expression Laboratory, Salk Institute for Biological StudiesLa Jolla, California, USA; 3 Center for Regenerative Medicine in BarcelonaBarcelona, Spain

**Keywords:** autophagy, cellular reprogramming, iPSC, stem cells, stemness

## Abstract

Stem cells have the ability to self-renew and differentiate into various cell types. Both cell-intrinsic and extrinsic factors may contribute to aging-related decline in stem cell function and loss of stemness. The maintenance of cellular homeostasis requires timely removal of toxic proteins and damaged organelles that accumulate with age or in pathological conditions. Autophagy is one of the main strategies to eliminate unwanted cytoplasmic materials thereby ultimately preventing cellular damage. Here, we shall discuss the accumulating evidence suggesting that autophagy plays a critical role in the homeostatic control of stem cell functions during aging, tissue regeneration, and cellular reprogramming.

## Introduction

Macroautophagy (hereafter referred to as autophagy) is a basic mechanism of degrading unnecessary or dysfunctional cell components. It is characterized by the engulfment of the targeted components in double-membrane bound autophagosomes followed by their fusion with lysosomes. Two ubiquitin-like conjugation systems are involved in the formation of autophagosomes: (1) ATG12 is covalently linked to ATG5 through ATG7, an E1-like enzyme, and ATG10, an E2-like enzyme; (2) microtubule-associated protein light chain-3 (LC3) is covalently linked to phosphatidylethanolamine (PE) through ATG7 and ATG3, an E2-like enzyme (Rubinsztein et al, 2011). Once autophagosomes are formed, their outer membranes fuse with lysosomes, with consequent disintegration of the inner autophagosomal membranes and degradation of the contents of autophagosomes by lysosomal enzymes. The produced catabolites include amino acids (AA), free fatty acids (FFA) and others, which are rapidly made available in the cytoplasm for recycling (Wirawan et al, 2012).

In somatic cells, the quality control of long-lived proteins and organelles is ensured by autophagy. Indeed, the autophagic process targets and degrades misfolded proteins or functionally impaired organelles thus preventing toxic effects due to their accumulation (Rubinsztein et al, 2011). The autophagic pathway can be activated by different stimuli including starvation, endoplasmic reticulum stress, DNA damage, and reactive oxygen species (ROS). The level of autophagic activity is tightly regulated through a number of signalling pathways (Egan et al, 2011; Rubinsztein et al, 2011).

While our knowledge of autophagy in somatic cell physiology is extensive, the role of autophagy in stem cells is much less understood. Recent studies have implicated autophagy in the homeostatic control and maintenance of the self-renewal capacity of stem cells. Additionally, autophagy may also participate in stem cell differentiation and somatic reprogramming (Vessoni et al, 2012). Under certain circumstances, autophagy can also trigger a cell death program termed autophagic cell death (Maiuri et al, 2007). In this Perspective, we mainly focus on the protective role of autophagy in various stem cell types.

## Autophagy in various stem cell types

**Figure 1:**
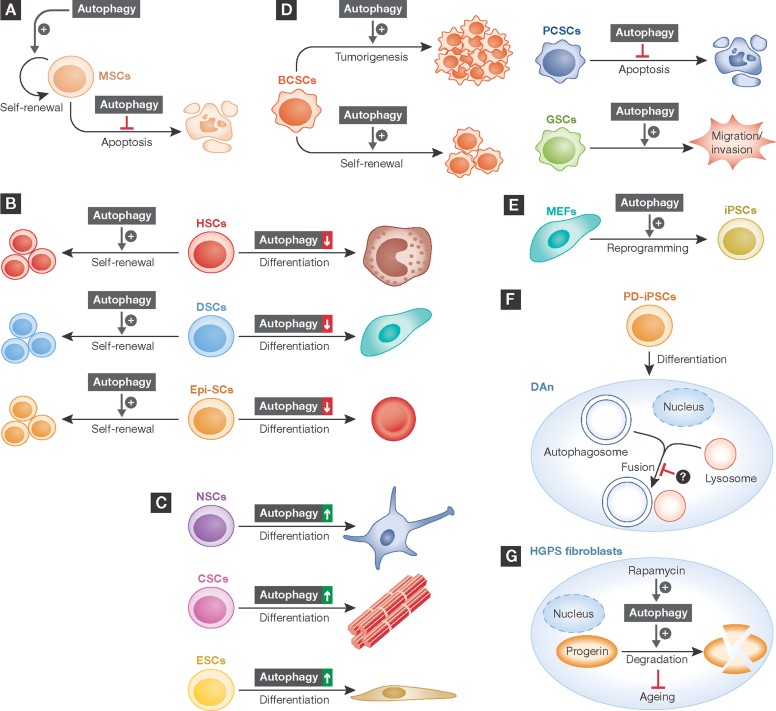
Autophagy in stem cell activity and function Autophagy is required for the maintenance of MSCs and inhibits their death.Autophagy remains at high levels in HSCs, DSCs and Epi-SCs, and promotes their maintenance; after induced differentiation, autophagic activity is down regulated.Autophagy in NSCs, CSCs and ESCs is up regulated during their differentiation.Autophagy is required for the maintenance and the tumourigenic potential of BCSCs, enhances the survival of PCSCs, and plays an important role in GSC migration.Autophagy increases the reprogramming efficiency, and promotes the generation of iPSC.Autophagosome clearance is inhibited in PD-iPSC-derived dopaminergic neurons (DAn).Rapamycin, an autophagy inducer, can effectively facilitate the degradation of progerin and thus prevent progeria-associated ageing phenotypes in Hutchinson-Gilford progeria syndrome (HGPS) fibroblasts.

A recent study has shown that the level of constitutive autophagy in human mesenchymal stem cells (hMSC) is high. Once hMSCs are differentiated into osteoblasts, however, basal autophagy becomes undetectable, suggesting that it is down regulated during hMSC differentiation (Oliver et al, 2012). Lee et al reported that autophagy induced by hypoxia promotes the maintenance and self-renewal of MSC (Lee et al, 2012; Fig 1A). Moreover, it was recently reported that activation of autophagy antagonized, while inhibition of autophagy promoted MSC apoptosis during hypoxia/serum deprivation (Zhang et al, 2012c; Fig 1A).

Autophagic activity has also been shown to be constitutively high in hematopoietic stem cells (HSC), dermal stem cells (DSC), and epidermal stem cells (Epi-S; Salemi et al, 2012). After induced differentiation, autophagic activity in immature keratinocytes, fibroblasts and neutrophils is down regulated to a basal level similar to that observed in most cell types (Salemi et al, 2012; Fig 1B). Other lines of evidence have shown that autophagy is required for the maintenance of HSCs (Liu et al, 2010; Mortensen et al, 2010, 2011a, b; Fig 1B). The specific deficiency of two essential autophagy genes FIP200 or Atg7, in mouse HSCs leads to their dysfunction and loss at the perinatal stage, and dysregulated myeloproliferation, suggesting a defect in self-renewal (Liu et al, 2010; Mortensen et al, 2011a).

At variance with MSCs, HSCs, DSCs and Epi-S', autophagy is up regulated during differentiation of neural and cardiac stem cells (NSC and CSC; Vazquez et al, 2012; Zhang et al, 2012a, b; Fig 1C). The expression of several main autophagy genes is increased in mouse embryonic olfactory bulb (OB) during the early stages of neuronal differentiation. Neurogenesis is markedly decreased when autophagy is blocked by chemicals (Vazquez et al, 2012). Neuronal differentiation is also impaired in Ambra1-null mice, Ambra1 haplodeficient cells and Atg5-null OB cells, further supporting the notion that autophagy plays a role in NSC differentiation (Vazquez et al, 2012). Similarly, inhibition of autophagy significantly inhibits CSC differentiation, while the same process is promoted by activation of autophagy (Zhang et al, 2012a, b).

Autophagy has also been reported to be rapidly up regulated during early differentiation of mouse embryonic stem cells (mESC) and human embryonic stem cells (hESC; Tra et al, 2011; Fig 1C). The deficiency of autophagy genes in mESCs during embryogenesis has long been studied (Mizushima et al, 2001). Null mutations of beclin1 in mESCs lead to early embryonic lethality (Yue et al, 2003), whereas impairment of autophagy caused by Ambra1 deficiency undermines the development of the nervous system (Maria Fimia et al, 2007). Moreover, deletion of Atg5 or Beclin 1 in mESC leads to compromised engulfment and clearance of apoptotic cells and the formation of defective embryoid bodies, further suggesting a critical role for autophagy in early embryonic development (Qu et al, 2007).

In addition to its functions in ‘normal’ stem cells, recent studies revealed that autophagy plays roles in cancer stem cells (also known as tumour initiating cells). Primary breast cancer stem cells (BCSC) have been shown to have a very high autophagic activity (Gong et al, 2012) and indeed knockdown of Beclin 1 and Atg7 in several BCSC lines leads to a significant impairment of self-renewal and a decline of tumourigenic potential (Gong et al, 2012) (Fig 1D). Another report suggested that autophagy could promote the survival of pancreatic cancer stem cells (PCSC; Singh et al, 2012; Fig 1D). In addition, autophagy also plays a significant role in glioblastoma stem cell (GSC) migration and invasion by modulating ATP metabolism and remodelling subcellular structures, such as regulating mitochondrial fusion (Galavotti et al, 2012; Fig 1D).

## Autophagy in somatic reprogramming

Autophagy has also been shown to participate in the regulation of the somatic reprogramming process. Indeed, pharmacological induction of autophagy increases the reprogramming efficiency of mouse embryonic fibroblasts (MEF) to induced pluripotent stem cells (iPSC; Chen et al, 2011; Fig 1E). These findings suggest the intriguing possibility that autophagy could serve as a positive regulator of induced pluripotency. The mechanisms through which autophagy might facilitate somatic reprogramming are not well understood. Autophagy might promote the induction of pluripotency by counteracting cellular senescence and apoptosis, both thought to be barriers to reprogramming (Menendez et al, 2011). Furthermore, ESCs have fewer mitochondria than their differentiated counterparts, which is consistent with the idea that mitochondrial oxidative phosphorylation leads to more ROS that may in turn impair long-term self-renewal of ESC (Armstrong et al, 2010). The autophagic degradation of mitochondria may ultimately improve the efficiency of reprogramming (Vazquez-Martin et al, 2012; Vessoni et al, 2012).

»…autophagy may serve as a critical mechanism for the regulation of self-renewal and differentiation.«

## Autophagy in iPSC-based disease models

Recent studies have established that autophagy could play significant roles in the pathogenesis of age-related diseases, especially neurodegenerative disorders (Hara et al, 2006; Harris & Rubinsztein, 2012; Shintani & Klionsky, 2004; Winslow & Rubinsztein, 2011). An accumulation of autophagic vacuoles was detected during the differentiation of iPSC generated from idiopathic Parkinson's disease (PD) and familial PD [associated with a mutation in the *Leucine-Rich Repeat Kinase 2* (*LRRK2*) gene] patients, to dopaminergic neurons (Sánchez-Danés et al, 2012). Moreover, induction of autophagy and/or inhibition of lysosomal proteolysis enhanced dopaminergic neuron phenotypic alterations, indicating that the defective maturation of autophagosomes into autophagolysosomes partially contributes to the LRRK2-associated PD phenotypes (Sánchez-Danés et al, 2012; Fig 1F). On the other hand, the stimulation of the autophagic pathway has been shown to slow down both physiological and premature aging processes. Induction of autophagy by rapamycin in Hutchinson–Gilford progeria syndrome (HGPS) fibroblasts can facilitate the elimination of progerin, the causative agent of accelerated cellular senescence, and thus the normalization of most progeria-associated cellular and molecular phenotypes (Cao et al, 2011; Fig 1G). Recently, three groups, including our own, have successfully generated iPSCs from progeria patients (Ho et al, 2011; Liu et al, 2011a, b; Zhang et al, 2011). Differentiation of progeria iPSCs into mesodermal tissues recapitulated the premature aging features after extended *in vitro* culture or under stress conditions. These defective iPSC derivatives will be valuable tools to study cell-type specific roles of autophagy in the development of cellular senescence and will also provide a platform to screen for the best autophagy regulators for potential pharmacological intervention of these accelerated aging disorders.

## Perspectives

The implication of autophagy in the maintenance of stemness adds a new layer of control on stem cell activity. Firstly, autophagy may serve as a critical mechanism for the regulation of self-renewal and differentiation. Indeed, stem cells require especially efficient protein turnover to eliminate unwanted proteins, which may otherwise accumulate and impair identity and function. Both autophagy and the ubiquitin-proteasome system (UPS) are important for protein quality control and the maintenance of cellular homeostasis, and they cooperate to regulate cellular aging (Jana, 2012; Rubinsztein, 2006). Dysfunction or decrease of the stem cell pools is typical of physiological and pathological aging; it would be therefore interesting to determine how these two protein degradation pathways are coordinated in the regulation of stem cell homeostasis, and how the dysregulation of autophagy in stem cells is linked to aging and degenerative diseases. Additionally, the involvement of autophagy in somatic reprogramming suggests a new methodological basis for developing strategies to efficiently generate iPSCs. Finally, increased autophagy may enable cells to overcome the cellular senescence barrier by remodelling the cell cycle machinery or by promoting the turnover of the ‘senescent’ subcellular architecture.

»…autophagy may enable cells to overcome the cellular senescence barrier by remodelling the cell cycle machinery or by promoting the turnover of the ‘senescent’ subcellular architecture.«

In summary, the study of the interplay between autophagy and cell stemness will not only increase our understanding of the mechanisms and pathways through which autophagy contributes to stem cell maintenance and differentiation, but also enhance our knowledge of the roles of autophagy in human development, aging, and various degenerative diseases (Sánchez-Danés et al, 2012). Stem cell rejuvenation and function and large-scale production of high quality transplantable materials through active manipulation of autophagic pathways using small molecules and/or targeted genome-editing technology may be more than a dream.
